# Associations between endothelial progenitor cells, clinical characteristics and coronary restenosis in patients undergoing percutaneous coronary artery intervention

**DOI:** 10.1186/s13104-018-3401-y

**Published:** 2018-05-08

**Authors:** Fernando S. Montenegro, Marcelo Correia, Fabiana Muccillo, Christina G. Souza e Silva, Andrea De Lorenzo

**Affiliations:** Instituto Nacional de Cardiologia, Rua da Laranjeiras, 374-Laranjeiras, Rio de Janeiro, 2224006 Brazil

**Keywords:** Endothelial progenitor cells, Coronary artery disease, Angioplasty, Restenosis, Angina pectoris, Acute coronary syndrome, Percutaneous coronary

## Abstract

**Objective:**

Endothelial progenitor cells (EPCs) are produced in the bone marrow and mobilized to the peripheral blood playing a key role in endothelial repair. The objective of this study was to evaluate circulating EPC before and after percutaneous coronary intervention (PCI) with stent implantation and their associations with coronary restenosis and adverse cardiovascular events. Venous blood was obtained before and the day after PCI. Quantification of total white blood count and identification of EPCs (CD45^−^CD34^+^CD31^+^CD133/2^+^CD309^+^) through immunophenotyping by flow cytometry was performed. The primary outcome was either restenosis detected by new coronary angiography or angina with myocardial ischemia at the territory of the stented coronary artery. Secondary outcomes were angina without demonstrable myocardial ischemia, acute coronary syndrome or all-cause death.

**Results:**

37 patients were followed for 1 year. The median EPC count before PCI was 320 cells/mcl and after PCI 286 cells/mcl. A decrease of EPC count was found in 65% of the patients, while 35% displayed an increase. Primary outcomes occurred in 10.8% and the secondary in 37.8% of the patients. Despite a higher level of EPC before (402 cell/mcl) and after PCI (383 cell/mcl) in patients with the secondary outcomes, there was no significant association between EPC and cardiovascular events.

**Electronic supplementary material:**

The online version of this article (10.1186/s13104-018-3401-y) contains supplementary material, which is available to authorized users.

## Introduction

Percutaneous coronary intervention (PCI) is currently one of the cornerstones of the treatment of coronary artery disease [1], at least in part due to the lower immediate morbidity and mortality rates in comparison to coronary artery bypass grafting [2]. However, early success may be hampered by restenosis, a reduction of coronary lumen diameter after PCI [3].

Angiographic restenosis is defined as a 50% narrowing at the PCI site in follow-up angiography, while clinical restenosis is considered present when ischaemic symptoms are seen and/or an ischaemic functional test is positive in individuals with ≥ 50% stenosis of the target vessel [4]. In earlier trials with bare metal stents, angiographic restenosis occurred in up to 1/3 of the procedures [5]. Drug-eluting stents (DES) allowed a reduction in the rates of angiographic restenosis by reducing neointimal hyperplasia; this led to their expanded use in more complex lesions and less selective populations, re-elevating the rates of restenosis. A recent study shows an incidence of target-vessel revascularization 1 year after PCI with DES of 7.8% [6].

Circulating endothelial progenitor cells (EPCs), first described by Asahara et al. [7], are bone marrow-derived cells that express a variety of endothelial surface markers and home to sites of endothelial denudation [8], with roles in endothelial repair and angiogenesis [9]. Endothelial damage induced by PCI can mobilize EPCs to the injured areas; the hypothesis is that, after weeks to months, these cells will proliferate and contribute to effective re-endothelization and the restoration of vascular homeostasis [10]. However, other studies have shown conflicting results of whether EPCs exert favourable or unfavourable effects at PCI sites [11]. Since further knowledge of the influence of EPCs on restenosis might help identify patients at higher risk of restenosis or future events [12], this study aimed to evaluate EPC counts before and after PCI and the associations between EPC numbers and both restenosis and clinical outcomes.

## Main text

### Methods

Male patients ≥ 18 years with stable angina scheduled for elective PCI with stent implantation at the National Heart Institute in Brazil between January and December 2014 were screened for the present study. Exclusion criteria included the presence of any acute or chronic inflammatory disease, recent surgery or trauma, malignant disease, chronic renal failure undergoing dialysis, acute coronary syndrome < 3 months, or any complication during PCI or in the 24 h post-PCI. The study was performed according to the Declaration of Helsinki and was approved by the local ethics committee (Comitê de Ética em Pesquisa do Instituto Nacional de Cardiologia). All participants gave written informed consent. The extent of coronary artery disease was quantified by (1) the number of coronary lesions determining > 50% obstruction, and (2) the number of coronary arteries with > 50% lesions. Hypertension was defined as a history of hypertension requiring antihypertensive therapy; a family history of coronary artery disease was defined as the presence of this disease in first-degree relatives (men < 55 and women < 65 years of age); diabetes mellitus was defined as the need for oral antidiabetic drug therapy or the use of insulin.

All biochemical analyses and flow cytometry studies were performed by an investigator blinded to patient data and outcomes. This was true for overall data analyses, interpretation and statistical analyses. Only the investigators who were responsible for patient recruitment and follow-up were aware of patient data and outcomes, but these investigators were not involved in the EPC analysis or the overall analysis of the results.

#### Flow cytometry

Peripheral venous blood was collected into 4-mL Vacutainer tubes (Becton–Dickinson, Basel, Switzerland) containing liquid tri-potassium ethylene diamine tetra-acetic acid (K3EDTA) immediately before PCI and the next day before hospital discharge (12 h after the procedure). Blood samples were refrigerated between 4° and 8 °C and processed within 24 h for total leukocyte count and for EPC isolation. EPCs were quantified by flow cytometry (BD FACSCanto; BD Biosciences, San Jose, CA, USA) and analysed using Infinicyt software (Cytognos, Salamanca, Spain). Briefly, EPCs were identified with phenotypes CD45+/−, CD34+, CD309+ and CD133+. We used a unique labelling and acquisition technique for flow cytometry, but all multiparametric analyses were performed in duplicate. The detailed EPC identification protocol can be found in Additional file 1.

#### Follow-up and outcomes

Patients were followed for 1 year, with clinical assessments every 3 months. The primary outcome was defined as the recurrence of typical or atypical angina and evidence of restenosis detected by coronary angiography or by evidence of inducible myocardial ischaemia in the region of the stented coronary artery, demonstrated by stress/rest myocardial perfusion scintigraphy performed according to standard protocols [13]. Secondary outcomes were typical or atypical angina without confirmation of ischaemia by myocardial scintigraphy, the occurrence of acute coronary syndrome (defined by typical symptoms and cardiac biomarkers elevated above the upper limit of normal or new pathological Q waves in at least 2 contiguous electrocardiogram leads) or all-cause death.

#### Statistical analyses

We estimated that 34 patients would be needed to provide an 80% power to detect a difference of 100 cells/mcl in the absolute EPC counts before and after PCI, with a standard deviation of 200 cells/mcl and an alpha level of 0.05.

Categorical variables are expressed as number and percentage and compared by Fisher’s exact test. Continuous variables were tested for normal distributions with the Kolmogorov–Smirnov test, are described as the mean ± standard deviation or median and interquartile range, and were compared by Student’s t test or Mann–Whitney’s test, as appropriate. Correlations were evaluated with Spearman’s test. For all analyses, R Commander statistical package was employed (Lucent Technologies, Murray Hill, NY, USA). A P < 0.05 was considered statistically significant.

### Results

#### Study population

Among 53 eligible patients, one was excluded due to post-PCI complications (atrial fibrillation) and 10 due to missing blood samples. Five patients were lost to follow-up, leaving 37 for analysis. The demographic and clinical characteristics and medications of the patients are listed in Table 1. Approximately 2/3 had multivessel coronary disease. All patients received at least one stent, most (83.7%) in only one coronary artery (35.1% in the left anterior descending coronary artery). The remaining 16.3% received stents in two coronary arteries. Bare-metal stents were placed in 44.2% of the patients, drug-eluting stents in 23.1, and 3.8% received both types of stents.

**Table 1:** Baseline characteristics

	n (%) or mean ± standard deviation
Age (years)	65.0 ± 8.2
Hypertension	34 (91.9%)
Diabetes	12 (32.4%)
Obesity	5 (13.5%)
Currently smoking	4 (10.8%)
Chronic kidney failure	3 (8.1%)
History of acute coronary syndrome	19 (51.4%)
Prior percutaneous coronary intervention	12 (32.4%)
Prior coronary artery bypass surgery	2 (5.4%)
Medications	
Aspirin	35 (94.6%)
Clopidogrel	25 (67.6%)
Angiotensin-converting enzyme inhibitors	6 (16.2%)
Angiotensin receptor blockers	21 (56.8%)
Beta-blocker	30 (81.1%)
Statin	31 (60.8%)
Number of coronary arteries with ≥ 50% obstruction	
1	12 (32.4%)
2	14 (37.8%)
3	11 (29.7%)
Number of stents (median)	2
EPC count before PCI (cells/mcl)	320

*EPCs* endothelial progenitor cell

#### Endothelial progenitor cells

**Fig. 1:**
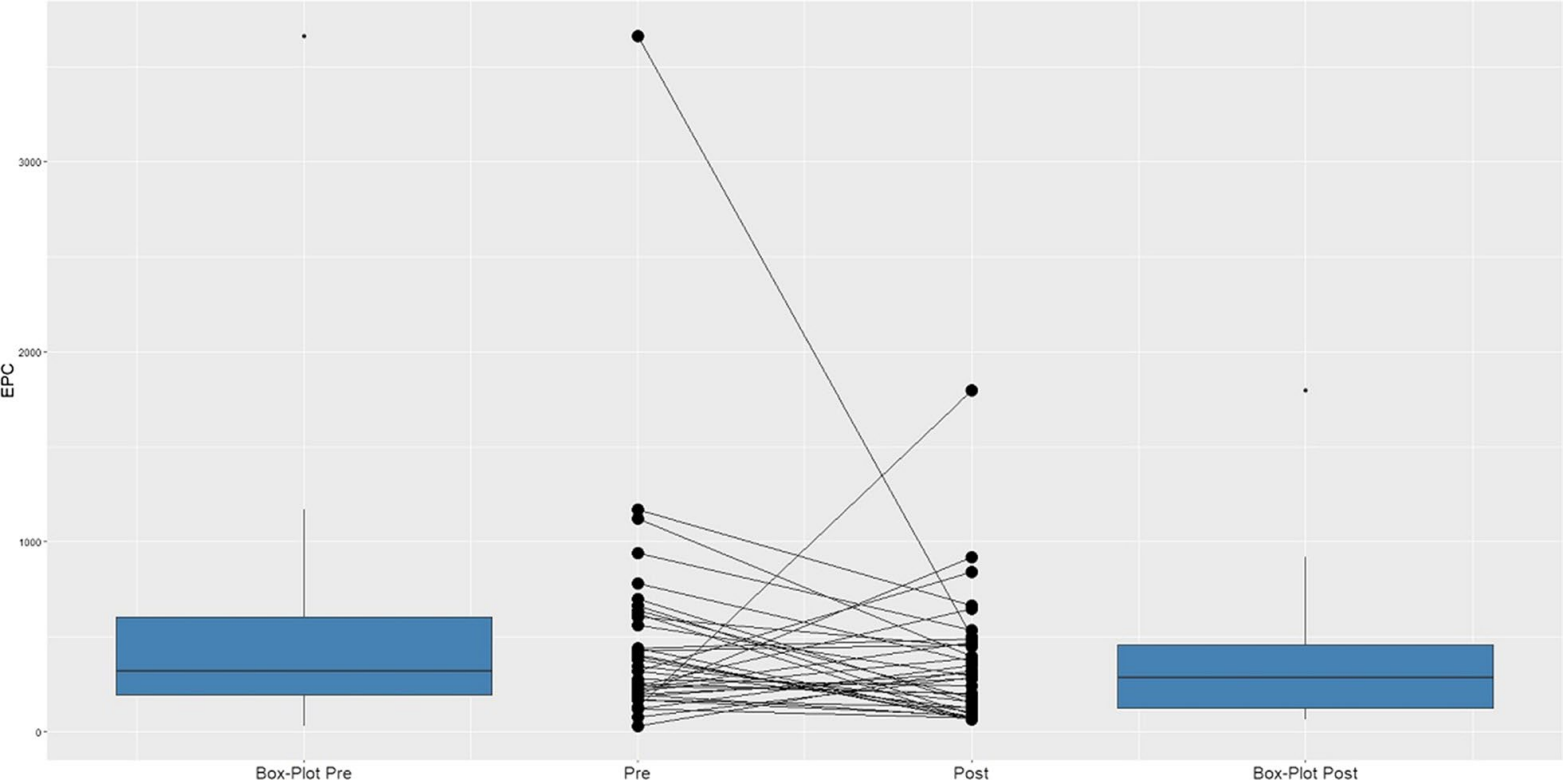
Changes in EPC counts before and after PCI. The boxplot on the left represents EPC counts before PCI with the median EPC count before PCI (320 cell/mcl). The boxplot on the right represents EPC counts after PCI with the median EPC count after PCI (286 cells/mcl). The middle graph shows the EPC behaviours for each patient

The median EPC counts before and after PCI were 320 and 286 cells/mcl, respectively (Fig. 1). Overall, a decrease in EPC count was found in 65% of the patients, while 35% showed an increase. The median pre-PCI EPC count, post-PCI EPC count and their difference, according to demographic characteristics and the presence of cardiovascular risk factors, are shown in Additional file 2: Table S1. None of the variables had any significant association with pre-PCI EPC count. There was no correlation between age and EPC count before or after PCI (Additional file 3: Figure S1). Post-PCI EPC counts were significantly associated with beta-blocker use (higher in patients taking beta-blockers). The difference between pre- and post-PCI EPC counts was higher in diabetic patients, who had a larger decrease in EPCs than nondiabetic patients (P = 0.04).

#### Outcomes and their associations with EPCs

Eighteen patients (48.6%) had one of the outcomes. Four patients (10.8%) showed primary outcomes; among them, three had restenosis confirmed by coronary angiography, and one had angina and inducible myocardial ischaemia. Secondary outcomes occurred in 14 patients (37.8%); of these, 10 had angina without evidence of inducible ischaemia, three had acute coronary syndrome, and one patient died due to a subarachnoid haemorrhage (Additional file 4: Figure S2).

Additional file 5: Figure S3 shows absolute the EPC counts in individuals with primary or secondary outcomes. Half of the patients with primary outcomes had an increase in EPC levels after PCI, and half a decrease (Additional file 5: Figure S3: Panel A). In patients with secondary outcomes, 64% showed an elevation in EPC counts after PCI, and 36% a reduction in EPC counts (Additional file 5: Figure S3: Panel B). There were no significant differences in EPC counts, pre-PCI, post-PCI or their difference, in patients with any of the outcomes (Table 2), even though the post-PCI medians were higher in patients with secondary outcomes.

**Table 2:** Comparisons of EPC counts in patients with or without primary or secondary outcomes

Outcomes	EPC count before PCI (cells/mcl)	P value	EPC count after PCI (cells/mcl)	P value	Difference in EPC counts	P value
Primary outcome (n = 4)	310 (230–442)	0.98	412 (157–715)	0.46	− 64 (− 485–322)	0.50
Without primary outcome (n = 33)	320 (192–602)		285 (124–450)		82 (− 82–361)	
Secondary outcome (n = 14)	402 (262–654)	0.17	383 (108–611)	0.34	231 (− 311–521)	0.55
Without secondary outcome (n = 23)	255 (189–499]		280 (136–369)		57 (− 102–296)	

Values are the median and interquartile ranges

### Discussion

Stent placement leads to mechanical injuries that induce substantial local inflammation, stimulating vascular smooth muscle cell proliferation and extracellular matrix depositions, resulting in neointimal thickening and restenosis [14]. After 3 months, complete reendothelization is noted [15].

The role of EPCs in the cardiovascular system and in the pathophysiology of coronary restenosis after PCI is a matter of debate. Although studies describe a protective effect of EPCs in the atherosclerotic process [12], in restenosis, there is a complex relationship between EPCs, endothelial repair and the induction of neointimal proliferation, such that it is still unclear if EPCs have beneficial or detrimental effects in this context [16].

Endothelial progenitor cell count is influenced by age and gender, explained in part by endogenous oestrogen levels [17, 18]. Therefore, due to the possibility of confounding, we decided to study only men, while being aware that this would limit the generalizability of the results.

Baseline EPC counts were inversely correlated with age and the extent of coronary artery disease (expressed by the number of coronary lesions), similar to that described by Schmidt-Lucke et al. [19].

Diabetic patients had a significant decrease in EPC counts, which parallels prior descriptions of decreased levels and reduced function of EPCs in patients with diabetes [20]. Lee et al. [21] investigated EPC mobilization after elective PCI in diabetic patients, demonstrating an absence of an increase in EPC levels. Similar results were found by Fadini et al. [22], showing EPC cell mobilization impairments in types 1 and 2 diabetics.

Prior studies have also demonstrated the impact of various pharmacological agents on the number and function of EPCs [23]. In this study, post-PCI EPC count was higher in patients taking beta-blockers. This is consistent with the results of Yao et al. [24] when using celiprolol, which increased the number of circulating EPCs and stimulated EPC colony formation and migration while decreasing EPC senescence.

Interestingly, in this study, a decrease in EPC counts after PCI was found in approximately 2/3 of the patients, which is different from other studies that have described increases hypothesized to result from focal EPC mobilizations due to endothelial injuries induced by PCI [25]. These different results can be explained, among other factors, by differences in the subgroups of EPCs studied and by differences in the degree of vascular injury induced by PCI.

Analysing patients who underwent elective PCI, Gao et al. [26] demonstrated different EPC mobilization behaviours in the 24 h after PCI depending on the degree of vascular injury induced by PCI. On the other hand, Thomas et al. [27] demonstrated a fall in EPC levels 6 h after the procedure.

Few studies have evaluated the relationship between EPC counts and restenosis or cardiovascular outcomes. In this study there was no significant association between EPC counts and outcomes after PCI. In the study by Pellicia et al. [28], patients with restenosis had higher EPC counts. However, in contrast to the present study, only bare-metal stents were used, cardiovascular risk factors were less common, and clopidogrel was used for only 1 month, which may explain the different results [28].

The study of EPC is challenging. There are no standard criteria for defining EPC, which may lead to the identification of different subpopulations of EPC [29]. Mobilization and function of EPCs are affected by cardiovascular risk factors, concomitant diseases and the amount of the endothelium that is damaged by PCI, making it difficult to control for all of these influences. Therefore, continued research is necessary to clarify the role of EPC in restenosis and outcomes after PCI, as these cells show promising effects when employed in stents [30].

## Limitations

The major limitation is the small sample size and short follow-up, which makes this a hypothesis-generating study. Additionally, the kinetics of EPC mobilization make it necessary to assess EPC more than one time after PCI. We did not assess the degree of endothelial injury after PCI, which can directly affect mobilization from the bone marrow. Finally, the use of fresh blood samples, longer time interval between pre-PCI and post-PCI, method of EPC measurement and definition of EPC are discussed in Additional file 6.

## Additional files

**Additional file 1.** EPC identification protocol.

**Additional file 2: Table S1.** EPC counts before PCI, after PCI and their difference.

**Additional file 3: Figure S1.** Boxplot figure correlating age with EPC counts before PCI and after PCI.

**Additional file 4: Figure S2.** Flowchart depicting patient outcomes.

**Additional file 5: Figure S3.** Changes in EPC counts before and after PCI.

**Additional file 6.** Limitations.

## Abbreviations

ACEi: angiotensin converting enzyme inhibitor
ARB: angiotensin receptor blocker
CABG: coronary artery bypass surgery
DES: drug eluting stents
EPC: endothelial progenitor cell
PCI: percutaneous coronary intervention
