# Supplementary material for: Associations between endothelial progenitor cells, clinical characteristics and coronary restenosis in patients undergoing percutaneous coronary artery intervention
Source: BMC Res Notes. 2018 May 8;11:278. doi: 10.1186/s13104-018-3401-y (PMC5941758; doi:10.1186/s13104-018-3401-y)
Supplement: Supplementary file 1 — Additional file 1. EPC identification protocol. [file 13104_2018_3401_MOESM1_ESM.docx]

**EPC identification protocol**

A panel of monoclonal antibodies was used, as follows: anti-CD45 / PerCP (5B1 clone, catalogue number 130.094.975, Miltenyi Biotec, Auburn, CA, USA), anti-CD34 / PE Cy7 (8G12 clone, catalogue number 348801, BD Biosciences, San Jose, CA, USA), anti-CD31 / FITC (WM59 clone, catalogue number 555445, BD Pharmigen, San Jose, CA, USA), anti-CD133 / PE (293C3 clone, catalogue number 130.090.853, Miltenyi Biotec, Auburn, CA, USA), and anti-CD 309 / APC (ES8-20E6 clone, catalogue number 120.006.197, KDR/VEGFR2; Miltenyi Biotec, Auburn, CA, USA).

Flow cytometric analysis was performed on whole blood samples without any enrichment procedures to avoid enrichment artefacts. Anticoagulated venous blood was aliquoted in 2.0 mL quantities into two 12 x 75 mm polypropylene test tubes (Becton Dickinson Labware, Franklin Lake, NJ, USA) and, after a gentle blending, incubated with 10 µL of each fluorochrome-conjugated MoAbs for 15 minutes at room temperature (RT) in the dark. The following directly conjugated mouse anti-human MoAbs were used for the first test tube: CD34 / PE Cy7, CD309 / APC, CD45 / PerCP, CD133/2 / PE and CD31 / FITC; no MoAbs were used in the second test tube for a blank control.

Stained whole blood samples were subjected to red blood cells lysis with 2 mL of lysing solution (BD FACS ^TM^ Lysing Solution - BD Biosciences, San Jose, CA, USA), vortexed and incubated for 15 min at RT in the dark. Prepared samples were stored in refrigeration between 4^0^ and 8^0^ C and analysed within 1 h. A total of 2 million events were acquired in 2 mL of whole blood, and these cells were analysed using sequential gating strategies that conformed to the guidelines of the International Society of Hematotherapy and Graft Engineering^1^. EPCs were defined as cells that were negative or “low” for haematopoietic marker CD45 and positive for markers CD34, CD133+, CD309+, excluding leukocytes (CD45+). We used a multiparametric analysis with sequential gating, employing Infinicyt software (Cytognos) to identify the phenotypes of the populations of interest, as follows: CD45^low^ or CD45- cells were selected, and the rest (CD45+) were excluded. Among the CD45^low^ or CD45- population, CD34+ cells were selected. Among the CD45^low^ or CD45- CD34+ cells, we identified those that were CD133+. Among the CD45^low^ or CD45- CD34+CD133+ cells, we selected those that were CD309+. We finally verified the distribution in a FCS X SSC histogram of the cells with the phenotype CD45^low^ or CD45- CD34+CD133+CD309+.

A sequential strategy was used to remove dead cells, platelet aggregates and debris and to exclude CD45+ cells, and then the sequence described above was followed^2^. The absolute number of EPCs was defined as the percentage relative to the white blood cell count assessed using a haematology cell analyser. EPC counts (pre-PCI, post-PCI and delta [calculated as pre-PCI count minus post-PCI count) are presented as number/microliter.

Internal quality assurance procedures were employed, including the daily calibration of flow cytometer optical alignment and fluidic stability using 7-Color SetUp Beads (BD Biosciences). The exactitude and stability of the cell counts were tested using internal quality controls and daily monitoring of whole-blood preparation procedures and MoAb reactivity using Immuno-Trol cells (Beckman Coulter, Fullerton, CA, USA).

References

1. Sutherland DR, Anderson L, Keeney M, Nayar R, Chin-Yee I. The ISHAGE guidelines for CD34+ cell determination by flow cytometry: International Society of Hematotherapy and Graft Engineering. J Hematother. 1996;5:213–226
2. Khan SS, Solomon MA, McCoy JP: Detection of circulating endothelial cells and endothelial progenitor cells by flow cytometry. Cytometry B Clin Cytom 2005, 64:1-8
