# Supplementary material for: Associations between endothelial progenitor cells, clinical characteristics and coronary restenosis in patients undergoing percutaneous coronary artery intervention
Source: BMC Res Notes. 2018 May 8;11:278. doi: 10.1186/s13104-018-3401-y (PMC5941758; doi:10.1186/s13104-018-3401-y)
Supplement: Supplementary file 2 — Additional file 2: Table S1. EPC counts before PCI, after PCI and their difference. [file 13104_2018_3401_MOESM2_ESM.docx]

**Table S1.** EPC counts before PCI, after PCI and their difference

| **Characteristic** | **EPC count before PCI**  **(cells/mcl)** | **EPC count**  **after PCI**  **(cells/mcl)** | **Difference in EPC counts** |
| --- | --- | --- | --- |
| Hypertension | 320 (192 – 591) | 282 (107 – 453) | 96 (-159 – 355) |
| Without hypertension | 438 (357 – 609) | 370 (305 – 429) | 36 (-7-223) |
| Diabetes | 482 (240 – 673) | 167 (115 – 302) | 296 (-159 – 355) ^*^ |
| Without Diabetes | 438 (357 – 609) | 370 (305 – 429) | 36 (-7 - 223) |
| Obesity | 243 (198 – 344) | 280 (204 – 396) | 39 (-82 - 186) |
| Without obesity | 320 (186 – 560) | 285 (102 – 455) | 57 (-172 – 340) |
| Previous smoking history | 361 (22 -633) | 337 (162 – 479) | 84 (-104 – 409) |
| Without smoking history | 259 (139 – 429) | 136 (79 – 284) | 139 (-49 – 322) |
| Chronic kidney failure | 438 (257 – 689) | 488 (386 – 511) | -50 (-129 – 177) |
| Without chronic kidney failure | 320 (193 – 591) | 260 (107 – 436) | 96 (-112 – 355) |
| Prior myocardial infarction | 320 (219 – 581) | 285 (167 – 479) | 57 (-127 – 304) |
| Without history of myocardial infarction | 349 (172 – 575) | 256 (82 – 389) | 96 (-99 – 398) |
| Prior PCI | 332 (199 – 470) | 277 (155 – 459) | 43 (-236 - 224) |
| Without prior PCI | 320 (192 – 621) | 285 (102 – 396) | 111 (-82 - 405) |
| Prior CABG | 349 (334 – 363) | 471 (286 – 655) | -122 (-321 – 77) |
| Without history of CABG | 320 (192 – 611) | 285 (126 – 452) | 82 (-102 – 383) |
| Aspirin | 320 (192 – 581) | 285 (126 – 452) | 82 (-102 – 350) |
| Without Aspirin | 444 (333 - 555) | 280 (184 - 375) | 164 (-42 – 370) |
| Clopidogrel | 395 (222 – 621)^*^ | 280 (129 – 396) | 196 (-50 – 411) |
| Without clopidogrel | 217 (158 – 346) | 297 (88 - 563) | 5 (-258 – 89) |
| ACEi | 214 (172 – 267) | 262 (213 – 303) | 37 (-82 – 71) |
| Without ACEi | 378 (207 – 611) | 291 (113 – 479) | 152 (-127 - 383) |
| ARB | 378 (216 – 621) | 158 (81 – 455)^*^ | 196 (-29 - 461) |
| Without ARB | 259 (181 - 570) | 329 (270 - 459) | 37 (-144 – 181) |
| Beta-blocker | 320 (193 – 628) | 329 (162 – 483)^*^ | 52 (-159 - 388) |
| Without beta-blocker | 378 (222 - 416) | 86 (73 - 171) | 276 (59 - 342) |
| Statin | 276 (192 – 581) | 309 (138 – 463) | 48 (-147 - 350) |
| Without statin | 332 (320 - 563) | 141 (97 – 171) | 191 (108 - 394) |

ACEi – angiotensin converting enzyme inhibitor; ARB – angiotensin receptor blocker; PCI – percutaneous coronary intervention; CABG – coronary artery bypass surgery. Numbers are median and interquartile range, * p< 0.05
