# Supplementary material for: Associations between endothelial progenitor cells, clinical characteristics and coronary restenosis in patients undergoing percutaneous coronary artery intervention
Source: BMC Res Notes. 2018 May 8;11:278. doi: 10.1186/s13104-018-3401-y (PMC5941758; doi:10.1186/s13104-018-3401-y)
Supplement: Supplementary file 3 — Additional file 3: Figure S1. Boxplot figure correlating age with EPC counts before PCI and after PCI. [file 13104_2018_3401_MOESM3_ESM.pdf]

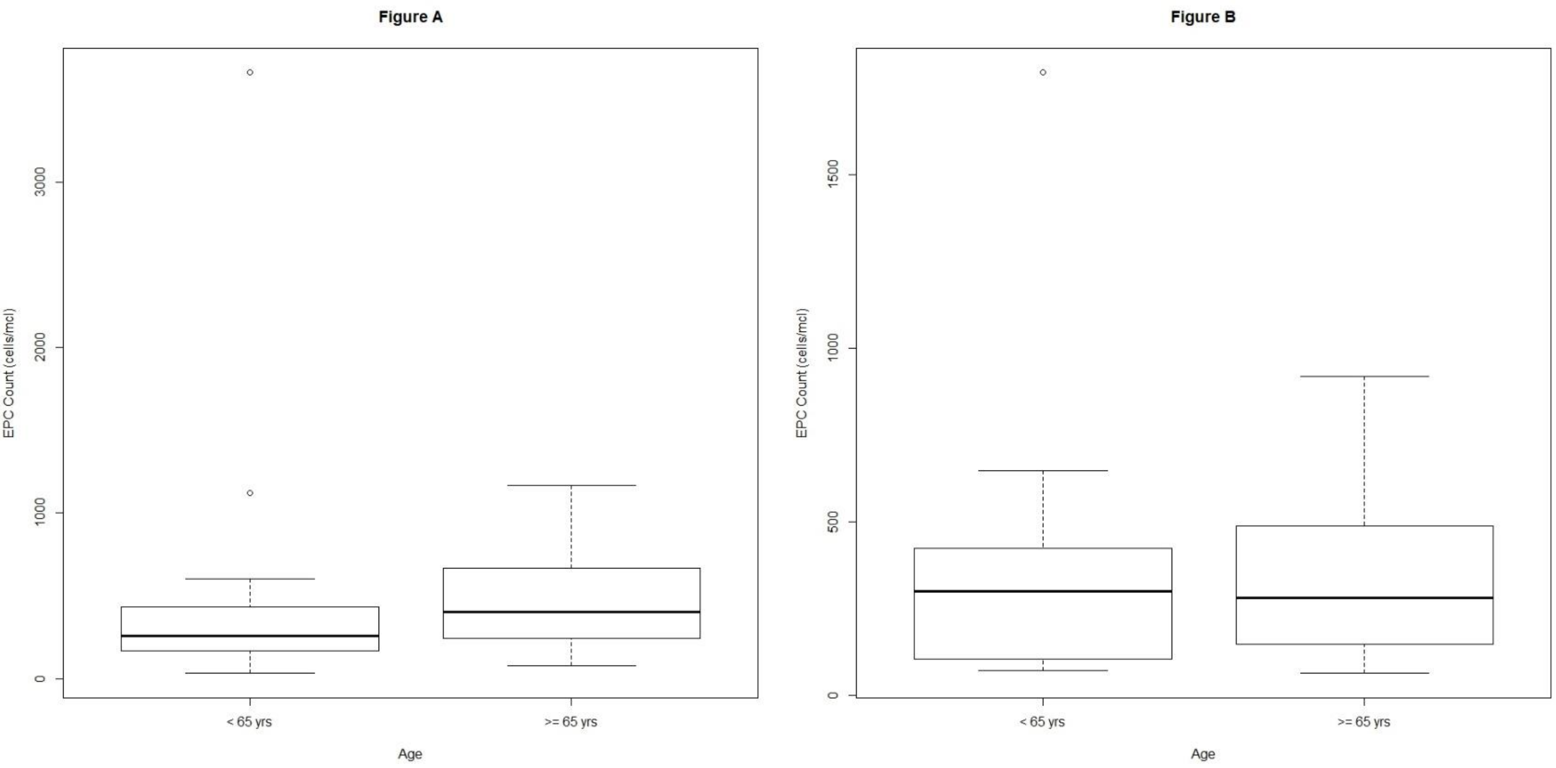

Figure S1: Boxplot figure correlating age (younger than 65 years and older than 65 years) with EPC counts before PCI (Panel A) and after PCI (Panel B). There was no correlation between age and changes in EPC counts after PCI.
