# Supplementary figures and images for: Associations between endothelial progenitor cells, clinical characteristics and coronary restenosis in patients undergoing percutaneous coronary artery intervention
Source: BMC Res Notes. 2018 May 8;11:278. doi: 10.1186/s13104-018-3401-y (PMC5941758; doi:10.1186/s13104-018-3401-y)

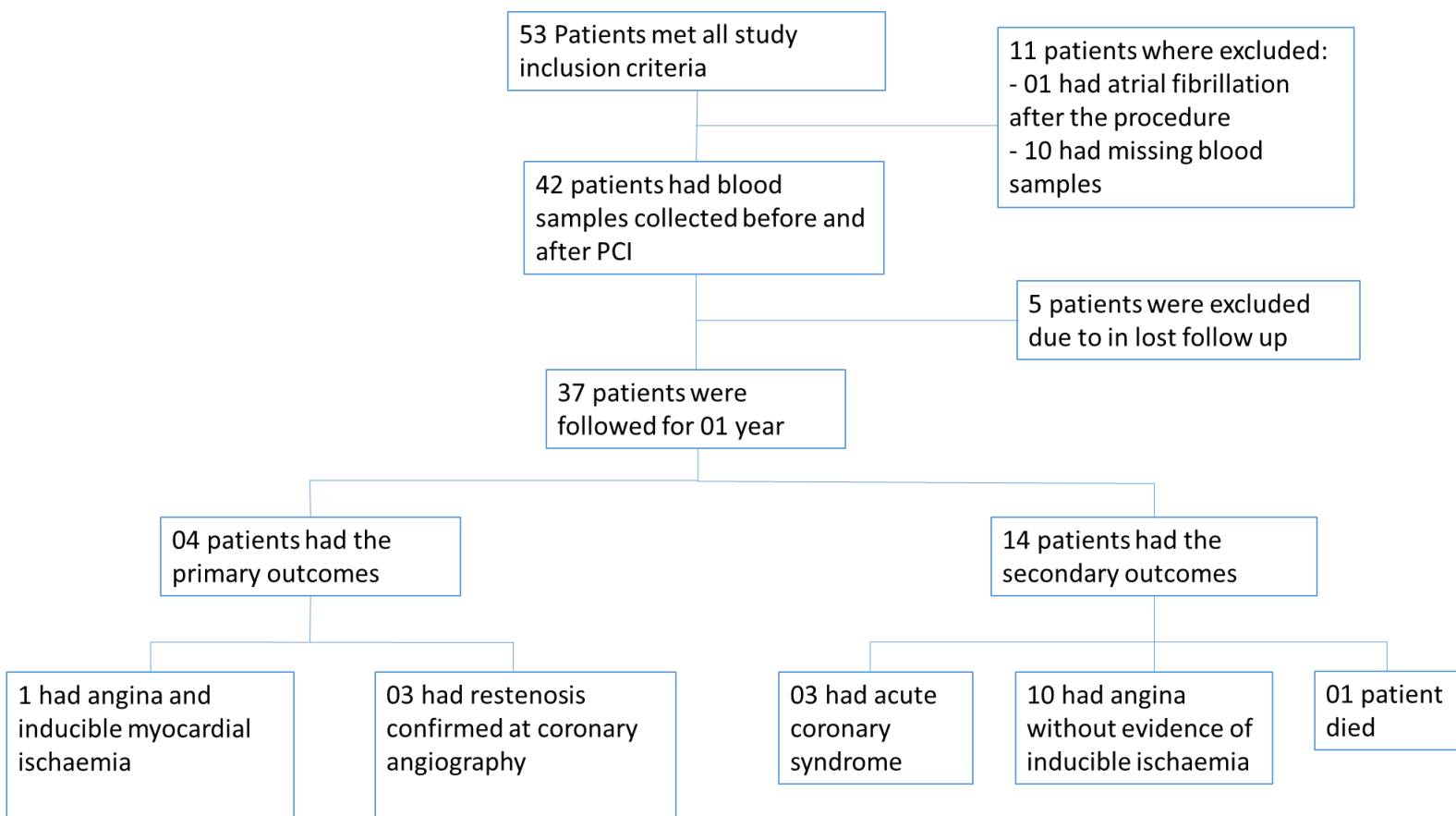

Figure S2. Flowchart depicting patient outcomes

Supplement: Supplementary file 4 — Additional file 4: Figure S2. Flowchart depicting patient outcomes. [file 13104_2018_3401_MOESM4_ESM.pdf]
