# Supplementary material for: Associations between endothelial progenitor cells, clinical characteristics and coronary restenosis in patients undergoing percutaneous coronary artery intervention
Source: BMC Res Notes. 2018 May 8;11:278. doi: 10.1186/s13104-018-3401-y (PMC5941758; doi:10.1186/s13104-018-3401-y)
Supplement: Supplementary file 5 — Additional file 5: Figure S3. Changes in EPC counts before and after PCI. [file 13104_2018_3401_MOESM5_ESM.pdf]

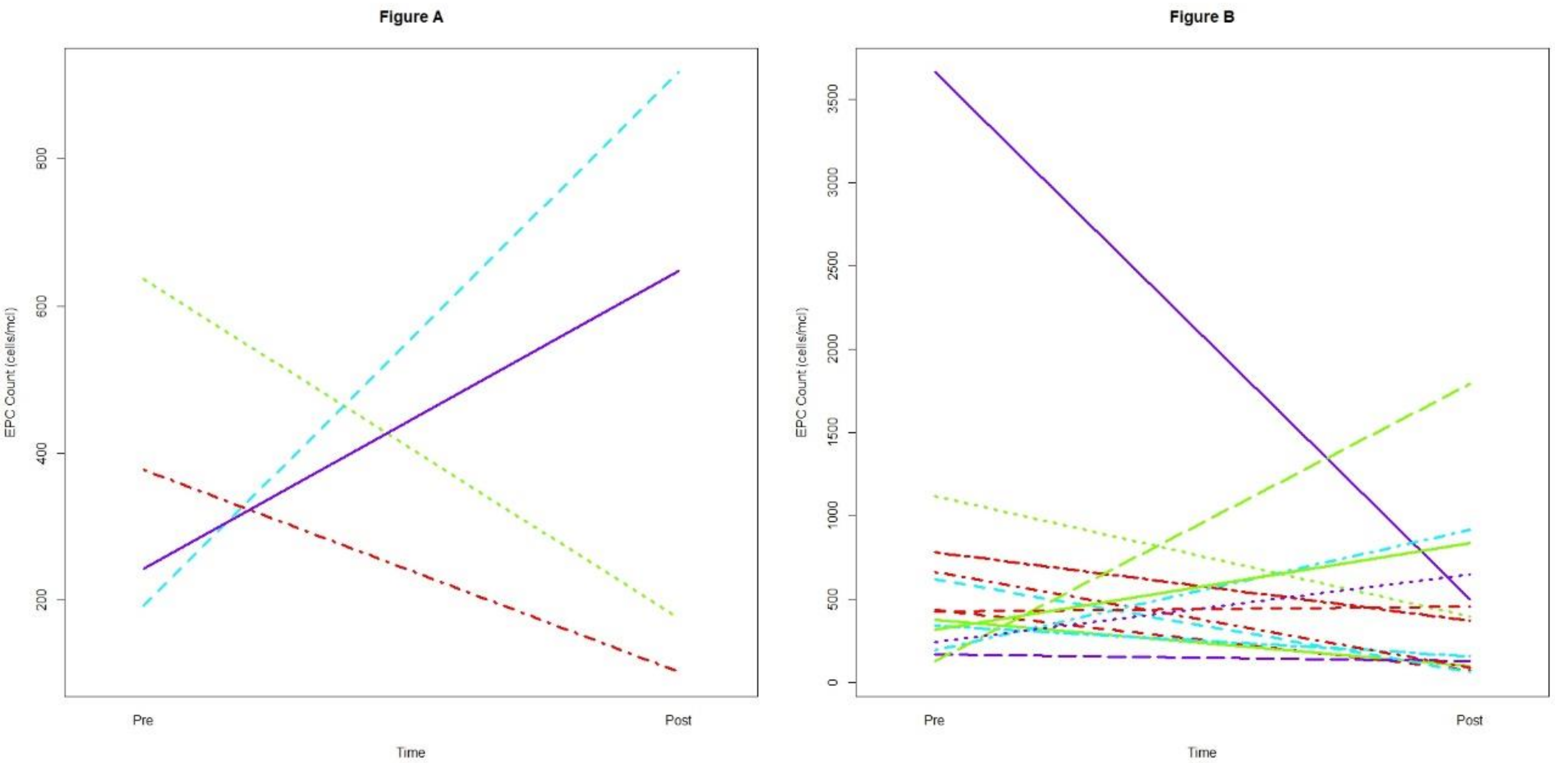


Figure S3. Changes in EPC counts before and after PCI. Each coloured line corresponds to a patient. Panel A: EPC counts before and after PCI in patients with primary outcomes. Panel B: EPC counts before and after PCI in patients with secondary outcomes.
