# Supplementary material for: Associations between endothelial progenitor cells, clinical characteristics and coronary restenosis in patients undergoing percutaneous coronary artery intervention
Source: BMC Res Notes. 2018 May 8;11:278. doi: 10.1186/s13104-018-3401-y (PMC5941758; doi:10.1186/s13104-018-3401-y)
Supplement: Supplementary file 6 — Additional file 6. Limitations. [file 13104_2018_3401_MOESM6_ESM.docx]

**Limitations**

Our study should be interpreted in the context of some limitations. Sample size, follow up, EPC mobilizations and degree of endothelial injury were discussed in the main text. In addition to these points, other important considerations will be listed below**.**

1. **It has been demonstrated that EPCs are unstable in extracorporeal blood samples. The use of fresh blood samples for EPC quantification may generate more consistent data.**

We agree that an important requirement for enumeration of EPC in clinical situations is stability of this cell population in stored blood samples. Masouleh et al measured EPC numbers in fresh blood samples and samples stored for 24 h and 48 h at room temperature (22 °C), respectively. Cell viability was stable at 24 h and 48 h after blood collection as determined by propidium iodide stains and trypan blue exclusion. However, the CD31+/CD133+ EPC counts decreased significantly after short-term storage for 24 h. In our study, all blood samples were processed within 24 hours reducing the likelihood of storage being a confounding factor in the EPC count^1^.

1. **There is only a 12-hr difference between the pre-PCI and post-PCI time points. It is felt that a longer time interval should be adopted to better reveal the relation between EPC and PCI.**

We agree that further blood sampling (ie, with larger intervals) might provide additional data on the relation between EPC and PCI. However, regarding EPC mobilization after percutaneous coronary intervention, Gao et al demonstrated different behaviors depending on the subpopulation studied. EPC characterized as CD34^+^KDR^+^ presented upregulation only after 24 hours of the procedure but EPC characterized as CD133^+^CD34^+^ increased their number within 7 hours of the procedure^2^. Bonello et al also demonstrated an increased number of circulating endothelial cells immediately after angioplasty with a peak 6 hours after the procedure^3^. This data suggests that a 12-hour interval could be enough to determine a difference in EPC levels before and after PCI depending on the subpopulation of EPC studied

1. **Measurement of CD34+/KDR+ cells may be an unreliable method for EPC enumeration. Instead, measurement of CD31+/CD133+ cells in a combined mononuclear cell and CD45-negative gating approach offers a reproducible method for EPC quantification.**

We understand that problems with the definition of EPC reflect the changing of surface markers as cells traffic from the bone marrow to the circulation and tissues, gradually losing monocytic and pan-leukocytic markers as they mature. Circulating, bone marrow-derived EPC have been described as CD45- or CD45low^4-6^. We did not assess EPC in cell cultures, but circulating EPC, which therefore may be CD45low or CD45-. The consensus combination of markers include CD34 and CD133^7^. Therefore, we used a multiparametric analysis with sequential gating, employing Infinicyt software (Cytognos) to identify the phenotypes of the populations of interest, as follows: CD45^low^ or CD45- cells were selected, and the rest (CD45+) were excluded. Among the CD45^low^ or CD45- population, CD34+ cells were selected. Among the CD45^low^ or CD45- CD34+ cells, we identified those that were CD133+. Among the CD45^low^ or CD45- CD34+CD133+ cells, we selected those that were CD309+. We finally verified the distribution in a FCS X SSC histogram of the cells with the phenotype CD45^low^ or CD45- CD34+CD133+CD309+.

1. **Measurement of EPC colony-forming unit (CFU) may be used to validate the findings and provide additional insight for the study.**

EPC colony-forming unit is another method of characterization based on in vitro behavior, including the ability to form endothelial colonies with the incorporation of acetylated low-density lipoprotein and biding of lectins. However, endothelial progenitor cells defined in these ways probably represent a mixed population, which, in combination with the lack of consensual definition, make the interpretation and comparison of woks in this field very difficult^8^.

References

1. Masouleh BK, Baraniskin A, Schmiegel W, Schroers R. Quantification of circulating endothelial progenitor cells in human peripheral blood: establishing a reliable flow cytometry protocol. J Immunol Methods. 2010; 357:38–42
2. Gao M, Yao Q, Liu Y, Sun F, Ma Y, Sun G, et al. Association between mobilization of circulating endothelial progenitor cells and time or degree of injury from angioplasty in patients with exertional angina: A prospective study. Exp Ther Med. 2015;10(2):809-815
3. Bonello L, Basire A, Sabatier F, Paganelli F, Dignat-George F. Endothelial injury induced by coronary angioplasty triggers mobilization of endothelial progenitor cells in patients with stable coronary artery disease. J Thromb Haemost. 2006;4:979–981
4. Goligorsky et al. Dysfunctional endothelial progenitor cells in chronic kidney disease. J Am Soc Nephrol 2010; 21:911-9
5. Steurer et al. Quantification of circulating endothelial and progenitor cells: comparison of quantitative PCR and four-channel flow cytometry. BMC Research Notes 2008; 1:71. doi:10.1186/1756-0500-1-71
6. Furstenberger et al. Circulating endothelial cells and angiogenic serum factors during neoadjuvant chemotherapy of primary breast cancer. British Journal of Cancer 2006; 94: 524 – 531
7. Goligorsky et al. Dysfunctional endothelial progenitor cells in chronic kidney disease. J Am Soc Nephrol 2010; 21:911-9
8. Leor J, Marber M. Endothelial progenitors: a new tower of Babel? J Am Coll Cardiol. 2006;48:1588-1590
